# Roles of frontal and temporal regions in reinterpreting semantically ambiguous sentences

**DOI:** 10.3389/fnhum.2014.00530

**Published:** 2014-07-29

**Authors:** Sylvia Vitello, Jane E. Warren, Joseph T. Devlin, Jennifer M. Rodd

**Affiliations:** ^1^Department of Experimental Psychology, University College LondonLondon, UK; ^2^Department of Language and Communication, University College LondonLondon, UK

**Keywords:** fMRI, LIFG, lexical ambiguity, speech comprehension, semantics, reinterpretation, sentence processing

## Abstract

Semantic ambiguity resolution is an essential and frequent part of speech comprehension because many words map onto multiple meanings (e.g., “bark,” “bank”). Neuroimaging research highlights the importance of the left inferior frontal gyrus (LIFG) and the left posterior temporal cortex in this process but the roles they serve in ambiguity resolution are uncertain. One possibility is that both regions are engaged in the processes of semantic reinterpretation that follows incorrect interpretation of an ambiguous word. Here we used fMRI to investigate this hypothesis. 20 native British English monolinguals were scanned whilst listening to sentences that contained an ambiguous word. To induce semantic reinterpretation, the disambiguating information was presented after the ambiguous word and delayed until the end of the sentence (e.g., “the teacher explained that the BARK was going to be very damp”). These sentences were compared to well-matched unambiguous sentences. Supporting the reinterpretation hypothesis, these ambiguous sentences produced more activation in both the LIFG and the left posterior inferior temporal cortex. Importantly, all but one subject showed ambiguity-related peaks within both regions, demonstrating that the group-level results were driven by high inter-subject consistency. Further support came from the finding that activation in both regions was modulated by meaning dominance. Specifically, sentences containing biased ambiguous words, which have one more dominant meaning, produced greater activation than those with balanced ambiguous words, which have two equally frequent meanings. Because the context always supported the less frequent meaning, the biased words require reinterpretation more often than balanced words. This is the first evidence of dominance effects in the spoken modality and provides strong support that frontal and temporal regions support the updating of semantic representations during speech comprehension.

## Introduction

Many of the words encountered in everyday language have multiple meanings, which makes the process of mapping word form onto meaning often ambiguous. This means that listeners must routinely combine various kinds of contextual information to understand the meaning that is intended by the speaker. For example, to understand the sentence “the woman used a microphone to make the toast,” listeners must use the word “microphone” to understand that the semantically ambiguous word “toast” refers to a celebratory speech rather than grilled bread. Importantly, such ambiguity is often not noticed by listeners (Rodd et al., 2005), suggesting that disambiguation is generally a highly efficient and effective process. An understanding of the neural substrates supporting this process is essential in order to gain insight into the efficiency of language comprehension and because the breakdown of this process can lead to severe communication difficulties due to the prominence of ambiguous words in everyday language (Parks et al., 1998; Rodd et al., 2002).

Cumulative evidence from recent neuroimaging studies has highlighted the importance of two brain areas for semantic ambiguity resolution: the left inferior frontal gyrus (IFG) and the left posterior temporal cortex (Rodd et al., 2005, 2010b, 2012; Davis et al., 2007; Mason and Just, 2007; Zempleni et al., 2007; Bekinschtein et al., 2011). However, the relative contributions of these regions to ambiguity processing are uncertain. Psycholinguistic research converges on several cognitive processes that underpin semantic ambiguity resolution: accessing the alternative meanings of an ambiguous word, selecting a single meaning, and reinterpreting that meaning when an incorrect selection is initially made (e.g., Duffy et al., 1988; Gernsbacher, 1991; Simpson, 1994; Twilley and Dixon, 2000; Duffy et al., 2001; Rodd et al., 2010a).

One hypothesis of the contribution of LIFG and posterior temporal cortex to ambiguity resolution is that both regions play an important role in reinterpretation processes (Zempleni et al., 2007; Rodd et al., 2010a,b; Bekinschtein et al., 2011). Semantic reinterpretation occurs when listeners encounter context that is not consistent with their initial understanding of the ambiguous word, requiring them to suppress the initially-selected meaning and integrate the alternative, contextually-appropriate interpretation (e.g., Duffy et al., 1988; Twilley and Dixon, 2000; Rodd et al., 2010a). Evidence for the reinterpretation hypothesis comes from the finding that activation in these frontal and temporal regions are greater for sentences with a higher likelihood of reinterpretation. For example, several functional MRI (fMRI) studies have shown increased activation in these regions for sentences in which the disambiguating information is delayed until after the ambiguous word (e.g., “The teacher explained that the BARK was going to be very damp”] compared to unambiguous sentences (Mason and Just, 2007; Zempleni et al., 2007; Rodd et al., 2010b, 2012; Bekinschtein et al., 2011). These are known as late-disambiguation sentences. Delaying the disambiguating information makes it impossible for listeners to determine the intended (e.g., tree) meaning of the ambiguous word when it is initially encountered. Thus, listeners will initially misinterpret the correct meaning on some occasions (i.e., first selecting the dog meaning of “bark”) and need to revise their understanding when they encounter the disambiguating information later on in the sentence (i.e., to the tree meaning). This process of initial meaning selection followed by reinterpretation is assumed in many influential cognitive models of semantic ambiguity resolution (Swinney, 1979; Twilley and Dixon, 2000; Duffy et al., 2001), on the basis of numerous cross-modal priming studies and eye-movement research which show that listeners and readers select a meaning within a few hundred millisecond of encountering an ambiguous word (e.g., Swinney, 1979; Seidenberg et al., 1982; Rayner and Duffy, 1986; Duffy et al., 1988). Various psycholinguistic studies, including eye-movement and dual-task research, also provide converging evidence that reinterpretation occurs for late-disambiguation sentences by showing that listeners and readers incur greater behavioral costs of processing the disambiguating regions in these sentences (e.g., longer reading times or poorer performance on an unrelated concurrent task) compared to processing equivalent regions in early-disambiguation sentences (e.g., “The hunter thought that the HARE in the field was actually a rabbit”) or unambiguous sentences (Rayner and Duffy, 1986; Duffy et al., 1988, 2001; Rodd et al., 2010a). Zempleni et al. (2007) provide more direct support that frontal and temporal regions support such reinterpretation processes by showing that activation in the LIFG and posterior middle/inferior temporal gyrus was modulated by meaning dominance, that is, how frequent the intended meaning is relative to the other meanings. Specifically activation in these regions was greater for late-disambiguation sentences that corresponded to the subordinate (i.e., less frequent) meaning than the dominant meaning (Zempleni et al., 2007). Reinterpretation is more likely in subordinate-biased sentences because people will typically select the dominant meaning in the absence of prior biasing context (Rayner and Duffy, 1986; Duffy et al., 1988; Simpson and Krueger, 1991).

Rodd et al. (2012) further proposed that the LIFG, in particular, may also be important for the initial selection of an ambiguous word's meaning that occurs when the word is initially encountered during a sentence (Twilley and Dixon, 2000). This suggestion was based on the finding that the LIFG, but not the posterior temporal cortex, was also more active for sentences in which reinterpretation was unlikely (compared to unambiguous sentences). These were sentences in which the disambiguating information *preceded* the ambiguous word. In addition, this region also showed activation that was temporally associated with the ambiguous word as well as the disambiguating information in late-disambiguation sentences. Thus, these results suggested that the LIFG may be involved in multiple processes of ambiguity resolution, not only when a meaning needs to be reinterpreted.

Supporting evidence for the involvement of the LIFG and posterior temporal cortex in reinterpretation and/or initial meaning selection, however, is not conclusive on various levels. First, the functional contributions of these regions to these two ambiguity-related processes is uncertain because not all studies have found the same response pattern to the different types of ambiguous sentences that load on these processes (Mason and Just, 2007; Bekinschtein et al., 2011). Second, different methods for examining neural responses to reinterpretation and/or initial meaning selection have been used for written sentences compared to spoken sentences. For example, studies have assessed how meaning dominance modulates ambiguity-related neural responses but these have only been conducted on visually-presented late-disambiguation sentences (Mason and Just, 2007; Zempleni et al., 2007). It is important to examine whether such dominance patterns found in the visual modality also replicate for spoken sentences in order to understand whether these ambiguity-related responses generalize across modalities. Although many of the regions reported in semantic ambiguity studies are considered modality-general (Binder et al., 2009; Novick et al., 2009; Price, 2012), it is possible that speech places different demands on ambiguity processes (particularly working memory aspects) due to the transient, fast-fading nature of the speech signal and, thus, may place different demands on the underlying neural circuitry. Third, the precise nature of the LIFG and posterior temporal cortex's involvement in ambiguity processes is also uncertain because there is considerable anatomical variability in the locus and extent of the ambiguity responses in these regions reported across studies (Rodd et al., 2005, 2010b, 2012; Mason and Just, 2007; Zempleni et al., 2007; Bekinschtein et al., 2011). As these anatomical differences relate to different anatomical regions that have been associated with different functions (see Price, 2012, for a recent review), it is important to explore the potential sources of this variability. It is possible that such variability reflects effects of statistical thresholds, differences in ambiguous stimuli or experimental protocols or even inter-subject functional variability given the finding of looser function-anatomy mappings for high-level cognitive processes (Duncan et al., 2009; Tahmasebi et al., 2012).

Furthermore, it is unclear how these ambiguity-responsive regions relate to those associated with sentence comprehension more generally. Do semantically ambiguous words place additional demands on regions that are already involved in the processing of sentences in general or do they engage regions that are more specific to semantically demanding stimuli? Neural models of language comprehension give different answers to this question. For example, Hagoort's unification account of LIFG function argues for the former, imputing a sentence-general function to this region (Hagoort, 2005, 2013), while Novick and colleagues' conflict resolution account argues for the latter (Novick et al., 2005, 2009). Such differences in perspective are also found across theories of the posterior temporal cortex's function in language processing (e.g., Hickok and Poeppel, 2007; Jefferies, 2013).

In summary, the current literature raises several questions regarding the involvement of the LIFG and posterior temporal cortex in semantic ambiguity resolution. What functional roles do these regions play in ambiguity resolution and sentence comprehension more generally? Which specific anatomical sub-fields within these regions are engaged by semantic ambiguity? How consistent is this ambiguity network across individuals? These questions were investigated using fMRI. Neural responses to a large set of late-disambiguation sentences were compared with those to well-matched unambiguous sentences. Based on previous research, it was predicted that ambiguity-elevated responses would be broadly found in the LIFG and the left posterior temporal cortex (Rodd et al., 2005, 2010b, 2012; Davis et al., 2007; Mason and Just, 2007; Zempleni et al., 2007; Bekinschtein et al., 2011). The areas showing a significant ambiguity effect were then investigated to answer three specific questions pertaining to function and inter-subject variability:

1. Are activations within these regions specific to ambiguous sentences or present for all sentences, albeit to a greater extent during ambiguous sentences? To address this question, unambiguous sentences were compared to a low level baseline condition. If regions showing an ambiguity effect support operations that are routinely involved in sentence comprehension then they should also show greater activation for unambiguous sentences compared to baseline. If, however, they support processes that are more specific to ambiguous stimuli, then they should not show an increased response to unambiguous sentences.
2. Is it possible to separate semantic reinterpretation processes from initial meaning selection in these regions by considering meaning dominance and are dominance effects for spoken sentences similar to those found for visually-presented sentences? Here dominance refers to the fact that some ambiguous words are biased in terms of the relative frequencies of their alternative meanings whereas others are balanced. Biased words such as “bank” have one more dominant meaning (i.e. financial institution rather than side of a river) whereas balanced words such as “bark” have meanings that are relatively equal in frequency (i.e., dog vs. tree). In this experiment, biased sentences were always disambiguated toward their subordinate meaning. By comparing responses to biased and balanced ambiguous words, we investigated the two key functions of ambiguity resolution: semantic reinterpretation and initial meaning selection. If regions showing an ambiguity effect are primarily involved in semantic reinterpretation, then responses should be greater for biased than balanced sentences because they have a stronger frequency bias that makes the inappropriate (dominant) meaning likely to be initially selected on most trials and, thus, need reinterpretation. In contrast, any regions that are primarily involved in initial meaning selection should show greater activation for balanced than biased sentences because this process is more difficult since listeners have less strong preferences for one particular meaning of these words (Duffy et al., 1988, 2001; Twilley and Dixon, 2000). If regions are involved in both processes (relatively equally), then they may show equivalently strong activation to both balanced and biased sentences, since they both load on (at least) one of these processes. In addition, two types of biased sentences were compared: strongly subordinate and weakly subordinate words. This comparison allows us to examine whether responses are merely related to the likelihood of reinterpretation, where the dominance pattern would be: “strongly biased” > “weakly biased” > “balanced,” or whether a less linear relationship exists between reinterpretation and ambiguity-responses. For example, a region may be especially engaged when very infrequent meanings need to be integrated, which would produce a pattern of: “strongly biased” > “weakly biased” = balanced”. See Table 1 for example sentences in each of the ambiguous and unambiguous conditions.
3. Finally, how consistent are these neuronal ambiguity-effects across individuals? Inter-subject variability of regions showing an ambiguity effect were assessed by examining whether the regions that showed reliable activation at the group-level were activated in all subjects.

**Table 1:** **Sentence conditions**.

**Sentence condition**	
Ambiguous (strongly-biased)	(e.g., The woman had to make the TOAST with a very old microphone)
Ambiguous (weakly-biased)	(e.g., The man was told that an ORGAN was not available for the choir)
Ambiguous (balanced)	(e.g., The teacher explained that the BARK was going to be very damp)
Unambiguous (control)	(e.g., The teacher explained that the steam was going to be very hot)

*In each example, the ambiguous word is capitalized and the disambiguating word is underlined*.

## Methods

### Participants

Twenty native monolingual British English speakers (11 female), aged 18–35 (*M* = 23.8) participated in the study. All were right-handed, had normal or corrected-to-normal vision and had no known hearing or language impairment. Participants were recruited from the UCL experimental subject pool and were paid for their participation. All gave informed consent and appropriate ethical approval was obtained from the UCL Departmental Ethics committee.

### Stimuli

Ninety two ambiguous auditory sentences were created based on items from Rodd et al. (2010a). They were all late-disambiguation sentences where the disambiguating context was presented after a semantically ambiguous noun. For example, in the sentence “the woman had to make the *toast* with a very old *microphone*,” “toast” is the ambiguous word (i.e., grilled bread vs. celebratory speech) and “microphone” is the disambiguating word. On average, the ambiguous words were presented 6.70 (*SD* = 1.00) words into the sentence and were a mixture of homographic and heterographic nouns (e.g., bark, night/knight). The disambiguation was always provided by the sentence-final word, except in four sentences where it was the last two words. There were at least 4 words between the ambiguous and disambiguating words (*M* = 5.79, *SD* = 1.46) to give listeners enough time to select their preferred meaning before they hear the disambiguating information (e.g., Swinney, 1979; Seidenberg et al., 1984; Rodd et al., 2010a). To ensure that the rest of the words were neutral, the sentences were created such that only the disambiguating word needed to be changed to instigate the alternative meaning. For example, in the above example, “microphone” could be replaced by “grill.” These alternative versions were not employed in the experiment.

To elicit semantic reinterpretation, the disambiguating words were chosen to correspond to the less frequent meaning of the ambiguous word because psycholinguistic research demonstrates that the dominant, (and in this case, incorrect), meaning is usually initially selected prior to disambiguating information (Duffy et al., 1988; Simpson and Krueger, 1991; Twilley and Dixon, 2000). The subordinate meaning was based on pre-test scores obtained by Rodd et al. (2010a). To validate these preferences, an independent group of 59 participants performed an extended version of the standard word association task typically used to measure meaning preferences (Twilley et al., 1994). Each ambiguous word was presented in isolation (e.g., fan) and participants typed the first related word that came to mind (e.g., wind, follower, cool). Because some responses could relate to more than one meaning (e.g., cool), after all the isolated words had been presented, the participants selected a definition of their intended meaning (e.g., admirer vs. ventilation device). This ensured that equivocal responses could be coded accurately (for further details see Vitello, 2014). A dominance score was subsequently calculated as the proportion of codable responses that were consistent with the meaning used in the experimental sentence (minimum 31 data-points per item). As expected, most words had low dominance scores (*M* = 0.25, *SD* = 0.20) indicating that the meaning used in the experimental sentences was the less preferred, infrequent meaning for the majority of items. These scores spanned across the four main categories of meaning dominance reported in the psycholinguistic ambiguity literature (Rayner and Duffy, 1986; Duffy et al., 1988; Sereno, 1995; Vuong and Martin, 2011): (1) 32 words were strongly subordinate-biased, where the meaning used in the experimental sentences was very infrequent, on average, preferred by only 6% of listeners (dominance range: 0–0.14); (2) 27 words were weakly subordinate-biased, where the sentence meaning was fairly infrequent, on average preferred by 21% of listeners (dominance range: 0.16–0.30); (3) 27 words were balanced, where the sentence meaning was one of two (or more) relatively equally frequent meanings, on average, preferred by 39% of listeners (dominance range: 0.31–0.54; (4) the remaining six sentences had high dominance scores, where the sentence meaning was, on average preferred by 77% of listeners (dominance range = 0.65–0.84). The range of “balanced” scores coheres with studies in which the less likely meaning of the balanced words was chosen (Rayner and Duffy, 1986; Sereno, 1995; Vuong and Martin, 2011). One-way independent-measures ANOVAs showed that the strongly-biased, weakly-biased and balanced conditions were matched on sentence-level properties (duration in seconds, number of syllables, number of words, position of the ambiguous word, position of the disambiguating word, naturalness rating; all *p*s > 0.2) as well as on lexical properties of the ambiguous word [log-transformed frequency, number of letters, number of meanings and number of senses, where “meanings” refers to semantically and etymologically unrelated meanings (e.g., bark) and “word senses” are semantically related (e.g., run), Rodd et al., 2002, all *p*s > 0.09].

Each ambiguous sentence was paired with a well-matched unambiguous sentence of similar syntactic structure that had a low-ambiguity noun in the position of the ambiguous word. For example, “the student had to wrap the *wrist* with a very old bandage.” Statistical tests confirmed that the ambiguous words had significantly more meanings and senses than the unambiguous words [*t*_(91)_ = 8.14, *p* < 0.001; *t*_(91)_ = 8.31, *p* < 0.001, respectively] (Online Wordsmyth English Dictionary-Thesaurus, Parks et al., 1998). They, however, did not differ significantly on other lexical properties, including log-transformed word frequency (CELEX lexical database, Baayen et al., 1995), and number of letters (all *p*s > 0.1) See Table 2 for corresponding descriptive statistics. The sets of ambiguous and unambiguous sentences were additionally matched on physical duration, number of syllables and number of words (all *p*s > 0.1). On average, both sets were also judged as highly natural, although statistically, the ambiguous sentences had lower naturalness ratings when rated on a 1 (highly unnatural) to 7 (highly natural) point scale by an independent group of 15 participants [*t*_(91)_ = 3.98, *p* < 0.001]. See Table 2 for sentence-level descriptive statistics. All sentences were spoken by the same female speaker (JMR).

**Table 2:** **Descriptive statistics [mean(*SD*)] for properties of the ambiguous and unambiguous target words and sentences**.

	**Property**	**Ambiguous**				**Unambiguous**
		**All**	**Strong biased**	**Weak biased**	**Balanced**	**All**
**TARGET WORD**	N	92	32	27	27	92
	Frequency (log-trans.)	3.61 (1.01)	3.66 (1.16)	3.82 (1.01)	3.24 (0.81)	3.63 (0.93)
	No. letters	4.72 (1.16)	4.50 (1.02)	5.04 (1.43)	4.56 (0.89)	4.76 (1.08)
	No. meanings	1.92 (0.90)	1.81 (0.64)	1.89 (0.97)	2.15 (1.10)	1.09 (0.32)
	No. senses	10.1 (5.60)	9.91 (4.79)	10.4 (7.03)	10.0 (5.06)	4.90 (3.09)
**SENTENCE LEVEL**	Length (seconds)	2.97 (0.29)	2.96 (0.25)	3.03 (0.30)	2.91 (0.34)	2.97 (0.31)
	No. syllables	16.5 (1.87)	16.3 (1.82)	16.9 (1.92)	16.1 (1.69)	16.4 (1.91)
	No. words	12.5 (1.23)	12.6 (1.34)	12.4 (1.18)	12.6 (1.15)	12.5 (1.23)
	Naturalness rating	5.46 (0.62)	5.37 (0.68)	5.60 (0.61)	5.40 (0.56)	5.80 (0.61)

Additionally, 46 filler sentences (50% ambiguous) were employed with the same structure as the experimental sentences. 14 were used in an initial practice block, 24 were catch sentences and the remaining 8 constituted dummy trials at the beginning of the fMRI runs. Catch sentences were presented with a visually presented probe word which participants had to decide was related or unrelated to the sentence. The aim of these catch trials was to ensure that attention was paid to the meaning of the sentences. Thus, for each catch sentence, a probe word was selected that was either clearly semantically related (50%) or clearly semantically unrelated (50%) to the sentence's meaning. Finally, to create a low-level auditory baseline condition, 32 experimental sentences were randomly selected and converted to signal-correlated noise (SCN) using Praat software (http://www.praat.org). Conversion to SCN involved replacing all the spectral detail with noise, rendering sentences unintelligible whilst maintaining low-level acoustic properties by retaining the original spectral and amplitude profiles. SCN was chosen as the baseline condition to be able to directly compare these results with those of previous fMRI studies on ambiguity (Rodd et al., 2005, 2012; Bekinschtein et al., 2011). An additional two sentences were selected and converted to SCN for the practice block.

The auditory stimuli were delivered over Sensimetrics insert earphones (http://www.sens.com/s14/) in the scanner. EQ filtering Software (Sensimetrics, Malden, MA, USA) was used to filter all sound files to ensure accurate frequency reproduction.

### Design and procedure

An event-related, within-subject design was employed in which participants were presented with all types of sentence trials (ambiguous, unambiguous, SCN and catch sentences) as well as silent (rest) trials. The rest trials were included as another baseline condition, having, on average, the same physical duration as the sentence trials (mean = 3 s; range: 2–4 s). The experiment was divided into four sessions, each with 70 trials: 23 ambiguous; 23 unambiguous; 8 SCN and 8 rest trials as well as two dummy trials to allow for T1 equilibrium before the test trials began. The stimuli were pseudo-randomized so that each run had an equal number of each stimulus type and no ambiguous sentence was placed in the same session as its matched unambiguous sentence in order to avoid potential syntactic priming effects. Each session lasted, on average, 8.47 min. The order of the sessions was counterbalanced across participants.

Each trial commenced with a white fixation cross in the center of a black screen. After 1000 ms, an auditory sentence stimulus or rest trial was presented. Then, for all trials, except catch trials, a silent period of 1500 ms occurred, followed by a jittered inter-trial interval (ITI) of 1000–3000 ms. For catch trials, a silent period of 500 ms followed the sentence offset, then the fixation cross was replaced by a probe word that was presented for 1000 ms on the screen (36 pt bold Helvetica font). Participants indicated whether the probe was related or unrelated to the sentence they just heard by pressing a button with the right index or middle finger. Response button order was counterbalanced across subjects. To discourage participants from actively waiting for a probe to appear and ensure attention to each sentence, we emphasized that responding to the probes would be straightforward if they listened carefully to each sentence. Participants practiced the task inside the scanner before the experimental blocks. The practice block contained a higher proportion of catch-trials than the experimental blocks so that participants could familiarize themselves with the task. A jittered ITI also followed the catch-trials sentences but this ranged from 2000–3000 ms to allow participants at least 3000 ms from probe-onset to respond and prepare for the next trial.

All stimuli were presented using MATLAB (Mathworks Inc.) and COGENT 2000 toolbox (www.vislab.ucl.ac.uk/cogent/index.html). The visual stimuli were projected onto a screen and viewed via mirrors mounted on the head coil. The auditory stimuli were delivered via MRI-compatible insert earphones (Sensimetrics, Malden, MA, USA, Model S-14), which provided a 20–40 dB attenuation level. Participants wore another set of ear protectors over the insert earphones to provide additional attenuation of the scanner noise. The experimenter checked participants could hear the sentences clearly over the noise of the functional EPI sequence prior to the experimental scanning blocks by conducting a practice run in the scanner.

### MRI acquisition

Participants were scanned at the Birkbeck-UCL Centre for Neuroimaging (BUCNI) on a Siemens Avanto 1.5T scanner. Whole-brain functional images were acquired with a gradient-echo EPI sequence (*TR* = 3000 ms; *TE* = 50 ms; 3 × 3 × 3 mm resolution). Each run consisted of 180 volumes. In addition, a high-resolution anatomical scan was acquired (T1-weighted FLASH, *TR* = 12 ms; *TE* = 5.6 ms; 1 mm^3^ resolution) for anatomical localization purposes.

### fMRI data analysis

The functional images were preprocessed and analyzed using Statistical Parametric Mapping software (SPM8, Wellcome Department of Cognitive Neurology, London, UK). Preprocessing involved realignment, spatial normalization and smoothing (8 mm FWHM Gaussian kernel) of the functional images. Entire datasets from three participants were removed because of excessive translational head motion (>3 mm) in at least three of the four scanning sessions. In a further participant, a single scanning session was excluded due to excessive head motion. Finally, for two participants the final five and seven volumes of one run were excluded due to motion. Spatial normalization combined an initial affine component with subsequent non-linear warping (Friston et al., 1995) to best match the Montreal Neurological Institute's MNI-152 template. The resulting images retained their original resolution (3 × 3 × 3 mm). Two analyses were conducted with separate general linear models. The first model combined all ambiguous sentences into a single condition regardless of ambiguous word dominance so that parameter estimates of the overall ambiguity effect would not be biased by the differences in sample sizes between the dominance conditions. At the first level, three experimental conditions (ambiguous, unambiguous and SCN) and one “dummy” condition that included the dummy sentences and catch-trials were modeled separately. For each trial, the onset of the sentence/SCN and its duration were specified. For the catch-trials 1.5s was added to the duration to incorporate the presentation of the visual word. Realignment parameters and temporal and dispersion derivatives were included as additional regressors to help model structured noise in the data. The derivatives, in particular, helped accommodate variability in the onset and duration of neural responses to the ambiguous sentences. At the group-level, random effects analyses were employed for two contrasts: “Unambiguous vs. SCN” and “Ambiguous vs. Unambiguous.” The former was conducted first to identify the general language network that is engaged under normal, low-ambiguity, speech. The latter identified the more specific ambiguity-elevated network. For each, the corresponding contrast parameter estimates for each subject were entered into the group-level analysis, where one-sample *t*-tests were computed. Activations were considered significant if they reached a threshold of *p* < 0.05 FWE corrected at the voxel level (Worsley et al., 1996).

The second analysis was identical to the first except that ambiguous sentence trials were modeled as separate dominance conditions. To achieve this, the first-level analysis model included four separate regressors corresponding to the four dominance conditions (strongly biased, weakly biased, balanced, and dominant). For each subject, parameter estimates were obtained for three contrasts: “Strongly biased > Unambiguous,” “Weakly biased > Unambiguous” and “Balanced > Unambiguous.” The dominant condition was not analyzed further due to the small number of trials in this condition. At the group-level, contrast images from these comparisons were entered into a One-Way repeated measures ANOVA to assess effects of dominance across the whole brain and were also employed in region-of-interest (ROI) analyses, described in more detail in the Results section.

Participants' structural images were normalized to the T1 template and a group mean structural image was created for data display purposes.

## Results

### Behavioral results

On the catch trials participants achieved a mean accuracy of 92% (range = 79–100%), with a mean reaction time of 1328 ms (*SD* = 345), indicating that all participants were paying attention to the meaning of the sentences.

### Unambiguous sentences vs. SCN

**Figure 1:**
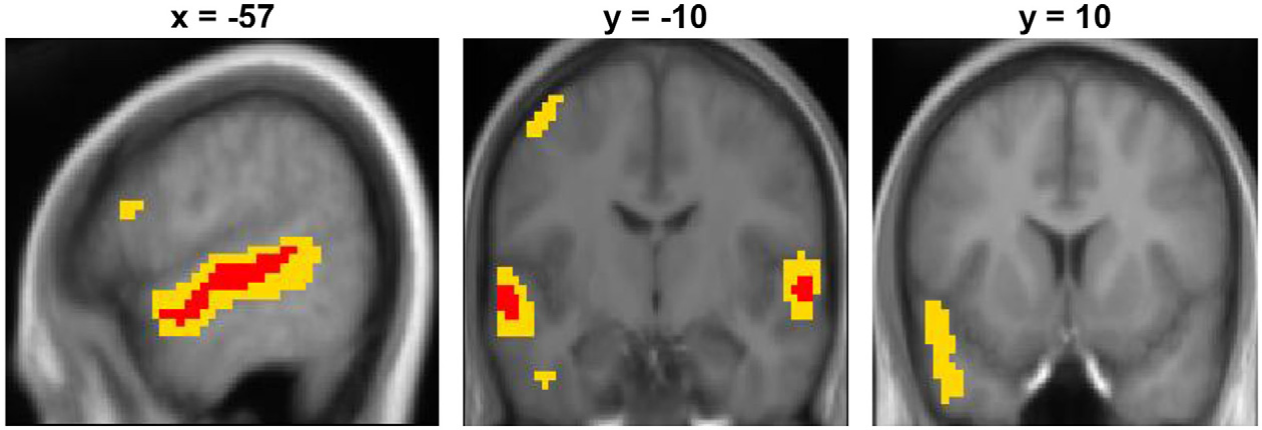
**Unambiguous sentence vs. SCN contrast displayed on the mean group structural image**. Red represents activation significant at *p* < 0.05 FWE-corrected and yellow represents activation significant at *p* < 0.001 uncorrected.

The contrast between unambiguous sentences and the low-level baseline condition showed a significant broad cluster of activation in the left hemisphere centered laterally on the mid-superior temporal sulcus (STS), extending along the length of STS and superiorly to the anterior superior temporal gyrus (STG) (see Figure 1 and Table 3). At a lower threshold (*p* < 0.001 uncorrected), the left anterior temporal activation spread inferiorly into anterior middle temporal cortex. In the right hemisphere, there was a smaller significant cluster of activation centered in mid STG that extended anteriorly into STG and STS. At the lower threshold (*p* < 0.001 uncorrected) it also extended posteriorly and inferiorly into right STS. There was also significant activation in the left dorsolateral precentral gyrus. The LIFG showed activation when the threshold was lowered to *p* < 0.001 uncorrected, specifically within dorsal pars opercularis (peak coordinate [−54, 17, 19]; *z*-score = 3.58). For completeness, the results of the Ambiguous > SCN contrast is presented in the supplementary materials (see Figure S1 and Table S1).

**Table 3:** **Unambiguous sentences > SCN: peak activations at *p* < 0.05 FWE corrected**.

**Brain region**	***p*(corrected)**	***Z*-Score**	**Co-ordinates (MNI)**		
			***x***	***y***	***z***
L STS	<0.001	6.11	−54	−25	−5
L anterior STS	<0.001	5.80	−57	−4	−14
L STS	0.001	5.58	−60	−16	−2
L posterior STS	0.011	5.08	−57	−40	7
R STG	0.001	5.60	60	−10	−2
R anterior STG/STS	0.030	4.88	60	−1	−11
Precentral gyrus	0.022	4.94	−48	−7	58

*Sub-peaks that are more than 8 mm from the main peak are indented*.

*L, left; R, right; STS, superior temporal sulcus, STG, superior temporal gyrus; MTG, Middle temporal gyrus*.

### Ambiguous vs. unambiguous sentences

**Figure 2:**
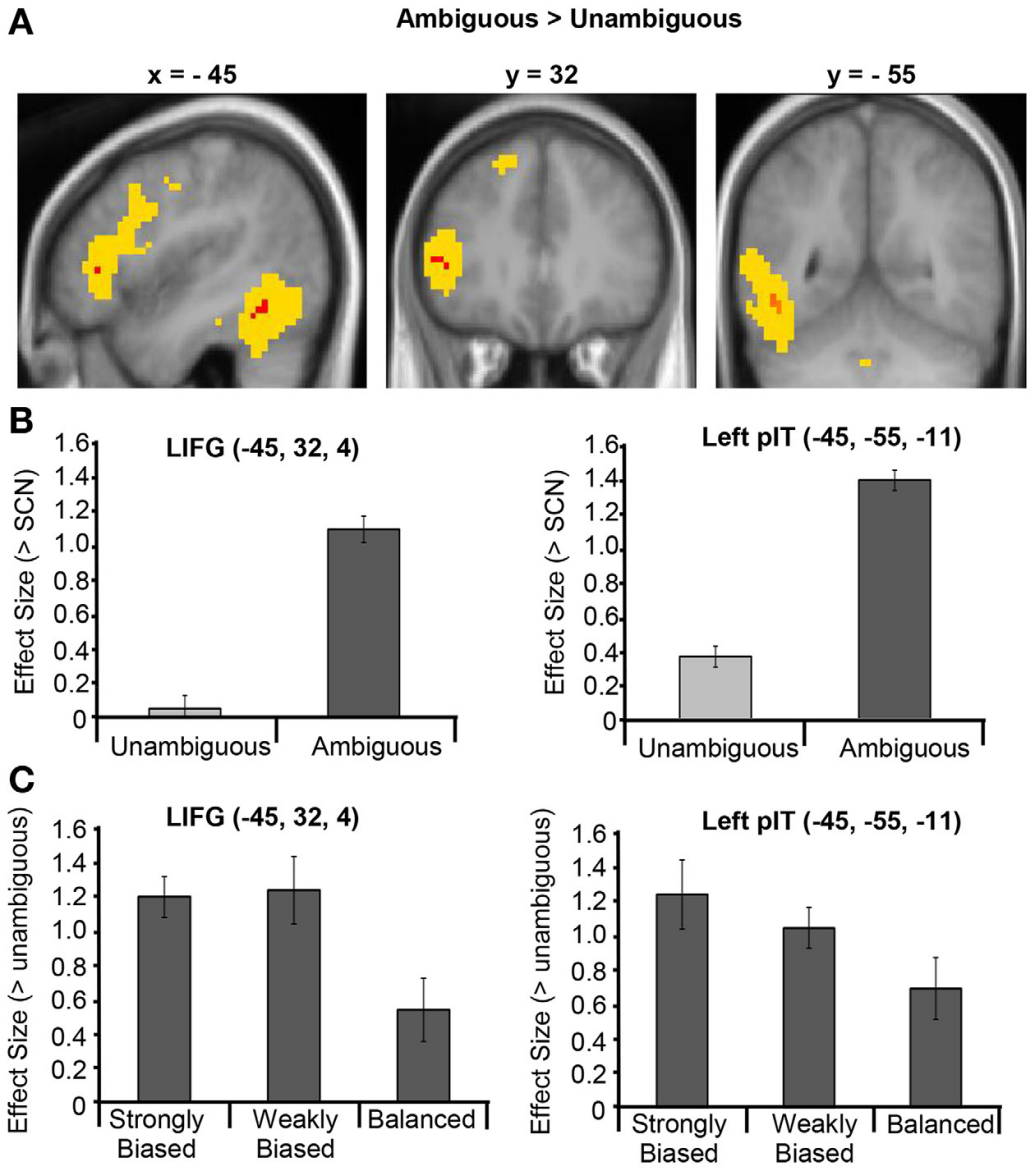
**(A)** Ambiguous vs. Unambiguous sentence contrast displayed on the mean group structural image. Red represents activation significant at *p* < 0.05 FWE corrected and yellow represents activation significant at *p* < 0.001 uncorrected. **(B)** Effect sizes for the Ambiguous > SCN contrast and Unambiguous > SCN contrast averaged across the LIFG and left pIT ROIs. **(C)** Effect sizes for the contrasts between each dominance condition and the unambiguous sentence condition averaged across the LIFG and left pIT ROIs. Error bars illustrate standard error on the means.

Two clusters in the left hemisphere showed significantly greater activation for ambiguous than unambiguous sentences (see Figure 2A and Table 4). One cluster was located in the LIFG, centered in pars triangularis. Note that this cluster does not overlap with the pars opercularis cluster that showed greater activation for the unambiguous sentences reported in the previous contrast. At a lower threshold (*p* < 0.001 uncorrected), the activation spread predominately posteriorly through pars opercularis, thereafter extending primarily dorsally in middle frontal/precentral gyrus. The second cluster was located in the posterior inferotemporal cortex (pIT). Its peak was in the posterior occipitotemporal sulcus (OTS) but extended laterally, with a significant sub-peak in the inferior temporal gyrus (LITG). At a lower threshold (*p* < 0.001 uncorrected) this activation extended inferiorly into the posterior and middle portion of the fusiform gyrus, as well as superiorly through the pMTG extending along the STS (see Figure 2A).

**Table 4:** **Ambiguous vs. unambiguous sentences: peak activations at *p* < 0.05 FWE corrected**.

**Brain region**	***p*(corrected)**	***Z*-Score**	**Co-ordinates (MNI)**		
			***x***	***y***	***z***
L IFG (pars triangularis)	0.027	4.90	−45	32	4
L OTS	0.011	5.09	−45	−55	−11
L ITG	0.012	5.06	−48	−58	−8

*Sub-peaks are indented following main peak*.

*L, left; OTS, occipitotemporal sulcus; ITG, inferior temporal gyrus; IFG, inferior frontal gyrus*.

The response profiles of the regions that showed a significant ambiguity effect were further examined with two region-of-interest (ROI) analyses, performed using the Marsbar toolbox within SPM8 (Brett et al., 2002). The first analysis assessed the nature of the ambiguity difference and the selectively of these regions' responses to ambiguous sentences by examining their responses to ambiguous and unambiguous sentences, separately, relative to SCN. For this analysis, a LIFG and a left pIT ROI were constructed as 8 mm radius spheres centered on the LIFG and left pIT group-peak coordinates obtained from the “Ambiguous > Unambiguous” contrast. Mean parameter estimates were obtained in each region for the contrasts “Ambiguous > SCN” and the “Unambiguous > SCN” for each participant. As shown in Figure 2B, the ambiguity difference in both regions was, importantly, driven by increased activity for the ambiguous sentences compared to SCN rather than deactivation in the unambiguous condition. In addition, one-sample *t*-tests revealed that neither the LIFG nor the left pIT ROIs showed a significant response for the unambiguous sentences compared to SCN [*t*_(16)_ = 0.17, *p* = 0.87; *t*_(16)_ = 1.88, *p* = 0.25, respectively].

A second ROI analysis assessed whether these regions were affected by meaning dominance. Mean parameter estimates for the strongly biased, weakly biased and balanced conditions relative to the unambiguous condition were obtained for the LIFG and left pIT ROIs. The resulting effect sizes for each region were normalized relative to the average effect size for that ROI across all participants and all three contrasts. This normalization adjusts for differences in overall effect sizes between ROIs that may confound the magnitude of the differences found between conditions between regions. The normalized effect sizes were entered into a 3 × 2 repeated-measures ANOVA with Dominance (strongly biased, weakly biased and balanced) and Region (LIFG, pIT) as the two factors. The results showed a significant main effect of Dominance [*F*_(2, 32)_ = 3.49, *p* = 0.04, η^2^_*p*_ = 0.18], no significant main effect of site and no significant Dominance x Region interaction (*F* < 1 in each case), indicating no reliable differences between the effect of dominance in the frontal and temporal regions. Paired *t*-tests between each pair of dominance conditions (averaged across region) showed that strongly biased sentences (mean = 1.23, *SD* = 0.80) and weakly biased sentences (mean = 1.15, *SD* = 0.77) produced significantly greater activation than balanced sentences [mean = 0.62, *SD* = 0.79: *t*_(16)_ = 2.21, *p* = 0.04; *t*_(16)_ = 2.19, *p* = 0.04, respectively). However, there was no significant difference between the strongly and weakly biased sentences [*t*_(16)_ = 0.35, *p* = 0.74]. See Figure 2C for the patterns of dominance effects for each of the ROIs.

No significant effects of dominance were found in the whole-brain analysis (*p* < 0.05 FWE corrected).

### Inter-subject variability

Although peak co-ordinates from the group analysis identify voxels that show the most reliable effects across subjects, it is also important to assess the inter-subject variability around these peaks. For each subject we obtained the nearest local maximum (*p* < 0.05 uncorrected) to the frontal [−45, 32 4] and temporal group activation peaks [−45, −55, −11] from the Ambiguous > Unambiguous contrast. The location was then examined on each subject's own structural image and identified according to sulcal landmarks. Only peaks that were within the frontal and temporal cortex were considered.

**Figure 3:**
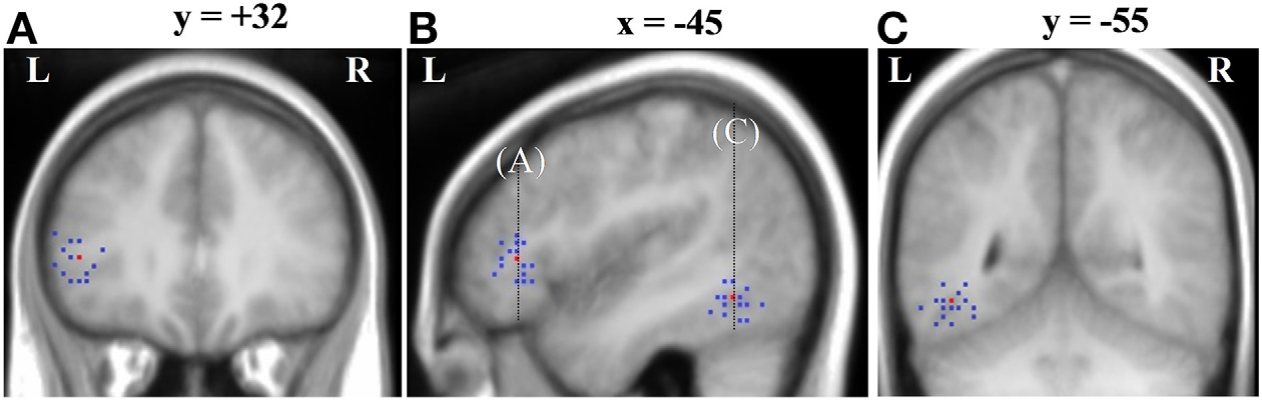
**Inter-subject variability around the Ambiguous vs. Unambiguous contrast group peaks displayed on the group mean structural image**. Red is the group peak and blue are individual subjects' peaks. **(A)** Variability around the LIFG group peak shown on a coronal slice where *y* = 32; **(B)** Variability around the LIFG and OTS group peak shown on a sagittal slice where *x* = −45; **(C)** Variability around the OTS group peak shown on a coronal slice where *y* = −55.

As shown in Table 5 and Figure 3, all subjects, except one, showed significant activation in close proximity to both the frontal and temporal group peaks. Only one subject did not show any significant activation around the frontal peak, with the nearest local maxima located 28 mm from the peak (coordinates [−21, 20, 13], *z*-score = 2.85). There was no significant difference between the two group peaks in terms of the average Euclidian distance of the local maxima [paired *t*-test: *t*_(15)_ = 1.37, *p* = 0.19]. Interestingly, the locations of these local maxima were notably more anatomically consistent (i.e., residing in the same macroanatomic region) in the frontal than in the temporal cortex. For 13 out of the 16 subjects who showed significant activation around the frontal peak, their local maxima resided in pars triangularis, with 2 additional subjects showing activation on the border between pars triangularis (PTr) and pars orbitalis (POr). In contrast, there was more anatomical variability around the temporal peak, with local maxima residing inferiorly within ventral occipital temporal cortex areas, such as OTS and fusiform gyrus (FSG), whilst others were located more laterally within MTG/ITG.

**Table 5:** **Individual subjects' “Ambiguous > Unambiguous” local maxima nearest to the frontal and temporal group peaks**.

**LIFG [−45 32 4]**							**L OTS [−45 −55 −11]**					
**Subject**	**x**	**y**	**z**	***Z* score**	**Distance from peak**	**Region**	***x***	***y***	***z***	***Z* score**	**Distance from peak**	**Region**
1	−36	35	7	2.20	9.9	IFS	−39	−52	−5	3.24	9.0	FSG
2	−45	32	10	2.94	6.0	PTr	−45	−49	−11	2.56	6.0	OTS
3	−45	29	−5	1.87	9.5	PTr	−48	−55	−5	2.57	6.7	MTG
4	−48	38	4	4.75	6.7	PTr	−48	−58	−11	2.06	4.2	ITG/OTS
5	−48	38	10	2.32	9.0	PTr	−45	−52	−14	2.53	4.2	OTS
6	−48	32	−5	2.62	9.5	POr/PTr	−45	−58	−20	3.53	9.5	ITG
7	−48	26	−5	2.54	11.2	PTr	−42	−67	−14	2.39	12.7	ITG/FSG
8	−39	38	1	4.26	9.0	PTr	−39	−52	−11	2.61	6.7	OTS/ITG
9	−54	29	1	2.53	9.9	PTr	−48	−52	−14	1.81	5.2	MTG/ITG
10			n/a				−36	−52	−17	1.76	11.2	FSG
11	−51	29	−2	2.84	9.0	PTr	−57	−61	−14	3.31	13.7	MTG
12	−48	29	10	2.52	7.3	PTr	−51	−61	−20	2.83	12.4	ITG
13	−54	32	13	1.87	12.7	PTr	−48	−46	−17	2.42	11.2	OTS
14	−54	26	1	3.62	11.2	PTr	−48	−58	−11	2.13	4.2	MTG
15	−51	32	7	2.07	6.7	PTr	−36	−58	−14	3.11	9.9	FSG
16	−42	41	−2	2.02	11.2	POr/PTr	−51	−58	−11	3.63	6.7	MTG
17	−42	29	−5	1.79	9.9	PTr	−42	−61	−8	1.82	7.3	FSG
Mean	−47	32	3	2.67	9.3 mm		−45	−56	−13	2.60	8.3 mm	

## Discussion

The results of this study replicate previous findings of increased activation in the LIFG and posterior temporal cortex for (temporarily) semantically ambiguous sentences compared to unambiguous sentences (Rodd et al., 2005, 2010b, 2012; Davis et al., 2007; Mason and Just, 2007; Zempleni et al., 2007; Bekinschtein et al., 2011). The current study employed ambiguous sentences for which listeners were likely to initially select the incorrect meaning of the ambiguous word and then need to reinterpret their understanding of the sentence later in the comprehension process. This was achieved by presenting the disambiguating information several words after the ambiguous word (e.g., “the woman had to make the TOAST with a very old *microphone*”), as various psycholinguistic models of ambiguity resolution claim that listeners make an initial meaning selection within a few hundred milliseconds of hearing an ambiguous word (Twilley and Dixon, 2000). Thus, this initial finding of ambiguity-responsive activation in the LIFG and posterior temporal cortex is consistent with the hypothesis that both of these regions may be important for reinterpreting the meaning of a word during sentence comprehension (e.g., Novick et al., 2005; Zempleni et al., 2007; Rodd et al., 2012).

This study, furthermore, explored the roles of these regions in ambiguity resolution by assessing their response profiles to different types of sentence stimuli as well as the inter-subject consistency of these regions' responses to ambiguity. The results of the functional-based analyses are discussed first, separately for the two regions, followed by discussion of the inter-subject variability.

Two specific functional questions were assessed. (1) Is activation within these regions specific to ambiguous sentences or present for all sentences, albeit to a less extent for low-ambiguity sentences? (2) Are these regions primarily contributing to semantic reinterpretation processes or initial meaning selection components of ambiguity resolution? For these questions, two contrasts were assessed via ROIs around the frontal and temporal group peak co-ordinate separately: (1) the regions' response to unambiguous sentences compared to a low-level auditory baseline and (2) the modulation of these responses by meaning dominance (i.e., meaning frequency) by comparing biased and balanced ambiguous words. A region showing an ambiguity effect that is primarily involved in semantic reinterpretation will show larger responses for biased than balanced sentences, whereas regions that are primarily involved in initial meaning selection will show the reverse profile. Together the results of these two contrasts give insights into the ways by which these regions support ambiguity resolution and language comprehension more generally, which ultimately help constrain theories of their functions in these processes.

### Left inferior frontal gyrus

Statistically robust activation (*p* < 0.05 FWE corrected) for semantically ambiguous sentences was found in the middle portion of the LIFG, namely pars triangularis (Figure 2A). This region has been reported in nearly all published studies on semantically ambiguous sentences (Rodd et al., 2005, 2010b, 2012; Davis et al., 2007; Mason and Just, 2007; Zempleni et al., 2007; Bekinschtein et al., 2011). Thus, this study corroborates it as the most consistent site of significant ambiguity-elevated peaks in the frontal cortex.

The results of the two additional contrasts showed two important findings pertaining to the role of this region in language comprehension. First, this region showed no significant response to unambiguous sentences compared to SCN (Figure 2B), suggesting that it may not be routinely involved during comprehension of low-ambiguity speech and may, therefore, perform different functions to those involved in general sentence processing. Several other neuroimaging studies have also failed to find significant LIFG responses to low-ambiguity sentences (Crinion et al., 2003; Spitsyna et al., 2006; Rodd et al., 2012).

This response selectivity for ambiguous but not unambiguous sentences is most consistent with the conflict resolution account of LIFG function (Thompson-Schill et al., 1997; Novick et al., 2005, 2009). According to this theory, the LIFG is involved in sentence comprehension only when there is conflict between simultaneously active representations in order to support the selection of one alternative. It is worth noting that although this region is not recruited by the relatively simple low-ambiguity sentences used in this study, its role is very unlikely to be specific to resolving semantic ambiguity as activation in this region has been observed for a range of other types of complex sentences including syntactically ambiguous sentences and syntactically complex sentences (e.g., Santi and Grodzinsky, 2010; Tyler et al., 2011).

The lack of a response for unambiguous sentences is less easily compatible with sentence-general accounts of the LIFG. For example, Hagoort's unification theory (Hagoort, 2005, 2013) proposes that the LIFG serves to combine small units of linguistic information into larger representations of a sentence. Therefore, all sentences should engage this region to some extent. Although the lack of an unambiguous response may merely be masked by activation in the baseline condition (Binder et al., 1999), patient data provide some corroborating evidence that the LIFG may not be necessary for and, thus, not always involved in language comprehension. For example, patients with LIFG lesions have relatively preserved comprehension of words and of relatively simple sentences (Caramazza and Zurif, 1976; Caplan et al., 1996; Yee et al., 2008; Novick et al., 2009).

It is important to note that the whole LIFG was not uniform in its response to the unambiguous condition. A more posterior region, in pars opercularis, showed greater activation for unambiguous sentences as well as an additional response to the ambiguous stimuli, although both of these effects were only significant at a more lenient statistical threshold. Thus, this suggests that there may be functionally distinct regions in the LIFG, some of which perform processes that are general to sentences and others that are more specific to certain types of sentences. However, it is not clear how this can be reconciled with claims that the function of the LIFG can be fractionated on the basis of either the linguistic nature of the processes (Gough et al., 2005; Vigneau et al., 2006; Hagoort, 2013) or the nature of the cognitive operation (Novick et al., 2005; Badre and Wagner, 2007).

The second key question concerned the effect of dominance (i.e., meaning frequency). The results revealed that mid-LIFG activation was greater for ambiguous sentences that contained a biased ambiguous word, which have one particularly dominant meaning (e.g., “toast”), than a balanced ambiguous word whose meanings are relatively equally frequent (e.g., “bark”; Figure 2C). This finding further supports the reinterpretation hypothesis, as listeners are more likely to reinterpret the meaning of a biased word because they were always disambiguated to their subordinate meaning (e.g., speech meaning of “toast”). Psycholinguistic research demonstrates that listeners and readers usually initially select the dominant meaning of a biased word when encountered before disambiguating context (e.g., the bread meaning of “toast”), whereas for balanced words there is less systematic bias for either alternative meaning across individuals (e.g., some may select the dog meaning of “bark” while others select the tree meaning). Thus, for biased sentences, the initial interpretation would often be incorrect and, hence, need to be reinterpreted more often than for balanced sentences. Although no significant dominance effects were found in the whole-brain voxel-wise analysis, this may reflect the fact that dominance responses are likely to be highly variable across both voxels and subjects, given the findings that meaning preferences are inherently variable across subjects (Rodd et al., 2013) and that the exact time-course of disambiguation varies across sentences (Rodd et al., 2012) and individuals depending on comprehension ability (Gernsbacher et al., 1990; Gernsbacher and Robertson, 1995).

The results of the dominance contrast directly replicate Mason and Just's (2007) finding of greater LIFG activation for biased than balanced sentences in visually-presented sentences, albeit in a more anterior ventral region, and provide the first evidence of these effects in spoken sentences. The results also converge with other dominance effects found in the LIFG, including greater activation for subordinate-biased compared with dominant-biased sentences (Zempleni et al., 2007) and the finding of a negative correlation between LIFG activation and the dominance of syntactically ambiguous sentences (Rodd et al., 2010b). Again, both of these effects reflect greater activation for sentences where reinterpretation is more likely. The finding that similar dominance effects in this region were found for this set of spoken sentences as has been reported for visually-presented sentences (Mason and Just, 2007; Zempleni et al., 2007) suggests a common system for disambiguating spoken and written sentences.

Interestingly, the current results showed that the two types of biased sentences patterned together: activation for strongly- and weakly-biased sentences was significantly greater than for balanced sentences but were not significantly different from each other. This suggests a non-linear relationship between dominance and neural response with neural responses not simply being associated with the likelihood of semantic reinterpretation. One possible reason for this pattern is that the neural responses may, in part, reflect how *difficult* reinterpretation is because this process is more demanding for biased than balanced words regardless of the extent of the bias *per se*. This explanation is derived from a large body of psycholinguistic research that demonstrates a difference in the state of the alternative meanings of biased and balanced words during the comprehension of late-disambiguation sentences. When biased words are encountered before disambiguating context, their dominant meaning is quickly integrated (e.g., the bread meaning of toast) while their subordinate (speech) meanings are quickly suppressed or not accessed at all. In contrast, multiple meanings of balanced words (tree and dog meanings of “bark”) are initially activated and it takes longer for one meaning to be integrated (e.g., Simpson, 1994; Twilley and Dixon, 2000; Duffy et al., 2001). Thus, contextually appropriate, subordinate, meanings may be harder to (re)integrate than non-selected balanced meanings because they are less available when the disambiguating information is later encountered and contextually-inappropriate, dominant, meanings may also be harder to override than initially-selected balanced meanings because they have been more strongly integrated (Simpson, 1994; Twilley and Dixon, 2000; Duffy et al., 2001; Gernsbacher and St John, 2001). Thus, the dominance pattern suggests that the LIFG may be particularly important to integrate less available meanings and/or suppress dominant incorrect representations during sentence reinterpretation. This is highly consistent with a recent patient study demonstrating that patients with damage to the LIFG had particular difficulty in resolving subordinate-biased sentences compared to sentences with balanced ambiguous words (Vuong and Martin, 2011). It is also compatible with Novick et al.'s (2005) view that the LIFG's role in sentence comprehension is to resolve misanalyses, although they refer to syntactic misinterpretations, and converges with findings in non-linguistic domains, such as emotion regulation where two recent meta-analyses have shown that the LIFG (and posterior temporal cortex) is engaged during reinterpretation of emotionally eliciting events (Buhle et al., 2013; Kohn et al., 2014).

This reinterpretation-based conclusion is predicated on the assumption that the greater activation for biased words is related to processes occurring at the time of the disambiguating information. It is therefore important to rule out alternative explanations that could potentially account for these effects in terms of processing at the time that they are initially encountered. At face value, such accounts seem unlikely as no current cognitive theories predict that there should be greater cognitive processing when encountering ambiguous words with one strongly dominant meaning compared with balanced words with two equally-frequent meanings, when these words occur in a neutral context. While it is, in theory, possible that biased words could induce greater processing demands if participants had learnt during the course of the experiment that when they encountered an ambiguous word they should interpret it with the less preferred meaning, existing behavioral and neuroimaging research strongly suggest that such expectations are either not learnt, or do not substantially influence, sentence comprehension. For example, numerous behavioral studies that have examined processing of these late-disambiguation sentences show that listeners' and readers do not experience behavioral processing costs (i.e., longer reading times or poorer performance on a secondary concurrent task) when they encounter biased ambiguous words in a sentence but only experience processing costs when the disambiguating information is encountered later in the sentence (e.g., Duffy et al., 1988, 2001; Rodd et al., 2010a). If biased words induced greater selection conflict at initial encounter with the word then such costs should be found at the time of the ambiguous word. In addition, even in fMRI studies where the ambiguity is concealed, such that participants do not report noticing any ambiguity in the sentences, activation is found in broadly similar brain regions, suggesting that such activity does not reflect greater selection conflict arising from an explicit strategy employed by the listener (Rodd et al., 2005).

In contrast to the current results, which emphasize the role of this region in reinterpretation, various theories suggest that the LIFG should also be important for processes associated with initial meaning selection (in the absence of reinterpretation) whenever this induces conflict (Thompson-Schill et al., 1997; Novick et al., 2009) and/or makes unification difficult (Hagoort, 2013). In addition, Rodd et al. (2012) found evidence that this region responds to both reinterpretation and initial meaning selection stages of ambiguity processing. Although the current results showed greater activation for sentences with a higher likelihood of reinterpretation, the results cannot rule out the possibility that it also responds to initial selection demands but the fMRI protocol was not sensitive enough to detect them. Future research would benefit from using techniques with higher temporal resolution than the fMRI protocol used here, as these processes occur at different times during sentence processing, such as magnetoencephalography (MEG) or time-sensitive fMRI techniques (Rodd et al., 2012), and should compare both the existence and magnitude of these responses.

In summary, the results replicate the involvement of the LIFG in ambiguity resolution and additionally show that this ambiguity-responsive region of the LIFG is not significantly engaged by all types of sentences to the same extent. This region shows no significant response to unambiguous sentences and demonstrates a larger ambiguity response for ambiguous sentences that are more likely to require reinterpretation. Together, the results are most consistent with accounts of this region that do not view LIFG as mandatory for language comprehension (e.g., conflict resolution account) and suggests that it supports comprehension when the listener's current interpretation needs to be updated in light of new contextual information.

### Posterior temporal cortex

In the temporal lobe, statistically robust activation for the semantically ambiguous sentences was located in the left posterior inferior temporal cortex (pIT), specifically in the occipitotemporal sulcus and inferior temporal gyrus. This is in a similar location to that found by Rodd et al. (2012) and Bekinschtein et al. (2011), but is more inferior than other studies where activation centers around pMTG/ITG (Rodd et al., 2005; Davis et al., 2007; Zempleni et al., 2007).

The results of the subsequent experimental contrasts showed that this region had a highly similar response profile to the mid-LIFG. The analyses showed (1) no significant response to unambiguous sentences (Figure 2B) and (2) the same pattern of dominance effects, where activation was greater for biased than balanced sentences (Figure 2C). Together, the results suggest that this region of the pIT is also involved in semantic reinterpretation processes which are not required for comprehension of low-ambiguity sentences.

The locus of this activation is interesting because it is posterior to regions more strongly associated with multimodal semantic processing, namely the anterior fusiform gyrus (Binder et al., 2009; Price and Devlin, 2011; Seghier and Price, 2011), and the cluster is more inferior than that associated with other lexical/semantic processes such as sound-to-meaning mapping in the pMTG/ITS (Hickok and Poeppel, 2007) and semantic control in the pMTG (Jefferies, 2013). Instead, this region has been more generally attributed to high-level visual processing associated with either the visual form of words (Dehaene and Cohen, 2011) or with visual features of meaningful stimuli more generally (Martin, 2007; Price and Devlin, 2011). This region is not consistently found in auditory single word or spoken sentence studies (Binder et al., 2000; Xiao et al., 2005; Spitsyna et al., 2006; Davis and Gaskell, 2009; Obleser and Kotz, 2010), but a large body of research shows that the response of this region is strongly modulated by non-visual processes such as semantics and phonological information (Devlin et al., 2006; Song et al., 2010; Yoncheva et al., 2010; Twomey et al., 2011) and can be activated in the absence of visual information (e.g., Mellet et al., 1998; Price et al., 2003). Thus, activation in response to ambiguity may reflect top-down accessing of visual information related to orthographic representations and/or visual attributes of the objects referred to in the sentence.

This view makes no strong prediction about whether this response should also occur for unambiguous sentences since these kinds of sentences may also evoke visual information. Indeed a recent study has reported activation for low-ambiguity speech in this region (Rodd et al., 2012). The lack of a response to unambiguous sentences, however, is incompatible with accounts that claim that such visual activation is a fundamental component of semantic processing (Martin, 2007).

Ambiguous sentences may engage visual information processing in various ways. For example, the ambiguity may evoke a visual image of the ambiguous word or an image of the content of the ambiguous sentence, which is supported by a large body of research showing increased activation of visual processing areas during imagery tasks (D'Esposito et al., 1997; Mellet et al., 1998; Martin, 2007; Dehaene and Cohen, 2011). Alternatively, visual representations may be activated more automatically by the increased level of semantic competition induced by the ambiguous words (Gennari et al., 2007), given the evidence of inherent functional and anatomical connections between semantic and perceptual representations (Kherif et al., 2011; Price and Devlin, 2011).

While the locus of this temporal activation is most consistent with regions discussed in visual processing accounts, it must be emphasized that it is also close to regions imputed in other accounts of posterior temporal function. In particular, this region is just inferior to pMTG/ITS that is argued to support sound-meaning mapping (Hickok and Poeppel, 2007; Hickok, 2012). Thus, the finding of an ambiguity effect in the broad vicinity of this region may also be considered consistent with this account, as the mapping between sound and meaning is more uncertain for ambiguous than unambiguous words. Presumably, this mapping needs re-computing when the meaning of word is not supported by contextual information (Rodd et al., 2012), which is further supported by the finding that this region was affected by reinterpretation load. However, it is difficult to explain the lack of significant response for unambiguous sentences in this region if it supports such a fundamental aspect of speech comprehension. Instead, the temporal areas that showed responses to unambiguous sentences were located more superiorly, along the STG/STS and anterior MTG. This distribution is consistent with previous studies on speech comprehension, where activation for low-ambiguity speech is typically confined to superior/middle temporal cortex (Humphries et al., 2001; Spitsyna et al., 2006; Adank and Devlin, 2010; Obleser and Kotz, 2010) rather than extending into inferior temporal regions in the way that is typically seen for studies of ambiguity resolution (Rodd et al., 2005; Davis et al., 2007; Zempleni et al., 2007; Bekinschtein et al., 2011).

In summary, the results replicate the involvement of the posterior inferior temporal cortex in ambiguity resolution and further show that activation in this region is not present for low-ambiguity sentences and is particularly responsive to ambiguous sentences that require reinterpretation. Like the response of the mid-LIFG, the results are most consistent with accounts of this region that impute functions that are not mandatory for sentence processing (e.g., visual-based processes) and suggests that this region also supports comprehension particularly when listeners needs to update their understanding of a sentence in light of new contextual information.

### Inter-subject variability

As this study confirms, the involvement of both frontal and temporal regions in the processing of semantically ambiguous sentences is emerging as a highly consistent finding across fMRI studies. However, these results are based on group-level analyses, which do not indicate the extent to which this reflects a network in which all components are engaged by all subjects. To investigate this, inter-subject variability was assessed in relation to the frontal and temporal group peaks. All subjects, except one, showed ambiguity-related local maxima within 10 mm of the LIFG and posterior temporal cortex group peak. These findings provide evidence that the group-level results reflect activation patterns that are consistent across a majority of subjects, rather than being driven by large activations in only a small proportion of individuals.

Other interesting findings also came out of this analysis. First, the anatomical locations of the LIFG individual peaks were highly consistent, being located within pars triangularis in over 80% of subjects. This further highlights the potential importance of this particular LIFG sub-division in semantic ambiguity resolution. In contrast, the locations of the temporal peaks were more anatomically variable. While the majority of subjects showed peaks in inferior, as oppose to middle, temporal regions (ITG, occipitotemporal sulcus, fusiform gyrus vs. MTG), there was no clearly consistent anatomical field. Such anatomical inconsistency in the temporal cortex's response to ambiguity across participants might explain why different studies have reported activation in these different sub-regions. The nature of this inter-subject variability is currently unclear, although several possible explanations exist. MRI and post-mortem investigations of the morphology of the temporal lobe have found that various macroanatomical structures (e.g., the inferior temporal sulcus) are extremely variable across subjects (Ono et al., 1990; Kim et al., 2000). The posterior inferior temporal cortex in particular has also been observed to have less distinct cytoarchitectonic boundaries such that neurologists have reported difficulty in subdividing this region based on microcellular properties (von Economo, 2009). These findings suggest that the relationship between function and macroanatomically-defined regions might be less consistent in the region and, thus, across subjects. Recent fMRI has further shown evidence that higher-level cognitive processes, more generally, show looser function-anatomy mappings than lower-level cognitive processes (Duncan et al., 2009; Tahmasebi et al., 2012). Alternatively, it is possible that this inter-subject variability found in this study may reflect functionally different responses to the ambiguous sentences across subjects, such that subjects draw on different cognitive operations to resolve the ambiguity. Although the reasons for such variability are currently uncertain, these findings clearly show inter-subject consistency of both frontal and temporal regions in processing ambiguous sentences.

### Additional ambiguity-responsive regions

Inspecting the data at a lower statistical threshold revealed that ambiguity-elevated activations occurred across substantially larger clusters within the frontal and temporal cortex than that shown when applying stringent statistical threshold. The frontal cluster extended throughout pars triangularis and pars opercularis. However, interestingly, activation was not found in its most anterior sub-division, pars orbitalis. This is particularly noteworthy as anterior LIFG has been specifically attributed to semantic processing (Poldrack et al., 1999; Gough et al., 2005; Hagoort, 2005, 2013; Vigneau et al., 2006; Badre and Wagner, 2007). This result is not entirely unexpected as the response of anterior LIFG to semantically ambiguous sentences is the least consistent of the three sub-divisions, with only two studies reporting activation across all three sub-divisions (Rodd et al., 2010b, 2012). One potential explanation is that this region serves a specific semantic-related function that is not important for resolving all types of ambiguous sentences. For example, one current theory of the anterior LIFG is that it supports controlled semantic retrieval (Badre and Wagner, 2007). In these sentences, the disambiguating word may have constituted a sufficiently strong semantic cue to the correct meaning of the word that additional retrieval processes were not needed.

Another interesting observation was the notable extension of ambiguity-related activation into frontal and temporal regions that have been strongly implicated in phonological processing, namely the posterior and mid-STS as well as the posterior LIFG (Hickok and Poeppel, 2007; Rauschecker and Scott, 2009; Hagoort, 2013). Such activation may reflect a replaying of the heard sentence in an attempt to reanalyze the meaning of these sentences. Thus, these additional results may provide working hypotheses for both cognitive and neural models of ambiguity resolution. It is, also, possible that these less robust regions may reflect inter-subject variability in the processing of ambiguous sentences.

Together these findings replicate the involvement of the LIFG and posterior temporal cortex in semantic ambiguity resolution found in previous studies and further demonstrate that this network is highly consistent across individuals. The results, additionally, explored the potential roles of these regions in this process, supporting the hypothesis that both regions may be particularly important when listeners need to reinterpret the meaning of an ambiguous word during sentence comprehension.

### Conflict of interest statement

The authors declare that the research was conducted in the absence of any commercial or financial relationships that could be construed as a potential conflict of interest.
