# Supplementary material for: Roles of frontal and temporal regions in reinterpreting semantically ambiguous sentences
Source: Front Hum Neurosci. 2014 Jul 29;8:530. doi: 10.3389/fnhum.2014.00530 (PMC4114184; doi:10.3389/fnhum.2014.00530)
Supplement: Supplementary file 1 [file DataSheet1.DOC]

***Supplementary Material***

**Roles of frontal and temporal regions in reinterpreting semantically ambiguous sentences**

**Sylvia Vitello_1_^1^*, Jane E. Warren_2_^12^, Joseph T. Devlin_3_^1^, Jennifer M. Rodd^1^**_4_

^1^ Department of Experimental Psychology, University College London, London, UK

^2^Department of Language and Communication, University College London, London, UK

*** Correspondence:** Sylvia Vitello, Department of Experimental Psychology, University College London, London, UK, s.vitello@ucl.ac.uk

1. **Supplementary Figures and Tables**

## Supplementary Tables

**Supplementary Table 1. Ambiguous sentences > SCN: peak activations at p<0.05 FWE corrected. Sub-peaks that are more than 8 mm from the main peak are indented.**

|  |  |  | **Co-ordinates (MNI)** | | |
| --- | --- | --- | --- | --- | --- |
| **Brain region** | **p(corrected)** | **Z-Score** | **x** | **Y** | **z** |
| L STS | <.001 | 6.14 | -54 | -22 | -5 |
| L anterior STS | <.001 | 5.83 | -57 | -7 | -11 |
| R STG | <.001 | 5.74 | 60 | -7 | -5 |
| R STG/STS | .013 | 5.04 | 60 | -1 | -11 |
| L anterior temporal | .007 | 5.16 | -33 | 5 | -47 |

Note: L, Left; R, Right; STS, Superior Temporal Sulcus, STG, Superior Temporal Gyrus.

## Supplementary Figures


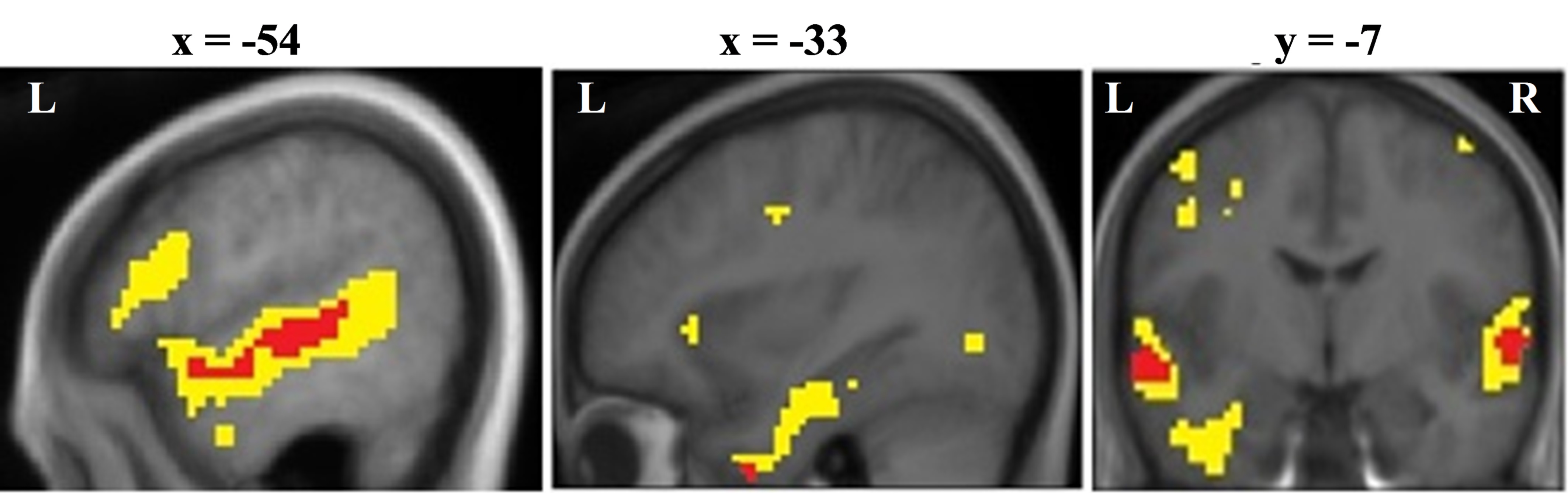


Supplementary Figure 1. Ambiguous sentence versus SCN contrast displayed on the mean group structural image. Red represents activation significant at p<.05 FWE-corrected and yellow represents activation significant at p<.001 uncorrected.
